# Mapmaking and Mapthinking: Cancer as a Problem of Place in Nineteenth-century England

**DOI:** 10.1093/shm/hky059

**Published:** 2018-10-08

**Authors:** Agnes Arnold-Forster

**Keywords:** cancer, mapping, nineteenth century, public health

## Abstract

In the nineteenth century, Dr Alfred Haviland plotted the distribution of cancer on maps of England. Matured within the intellectual milieu of nascent professional public health, his work can be married to that of his fellow sanitary reformers; however, his approach to medical cartography differed from what historians expect of Victorian mapmakers. While most of his mapmaking colleagues attended to urban places, Haviland turned his attention to the English countryside. This article will thus make three interventions into the limited literature on cancer in nineteenth-century England. First, it will demonstrate how cancer came to be constituted as a problem of place. Second, it will show that Haviland understood the disease to be produced by rural environs, and thus paradoxically correlated to healthful locales rather than areas of urban squalor. Third, this article suggests an alternative to the well-travelled interpretation of nineteenth-century mapping as an exercise in power and social control.

In the nineteenth century, Dr Alfred Haviland plotted the distribution of cancer on chloropleth maps of Britain.^1^ Haviland was devoted, like many of his mapmaking peers, to organising the country’s health, wealth and well-being by arranging it on the printed page.^2^ Matured within the intellectual milieu of nascent professional public health, his work can be married to the actions and intentions of his fellow sanitary reformers; however, his approach to social and medical cartography differed from what historians have come to expect of Victorian mapmakers. While most of his mapmaking colleagues attended to urban places, Haviland turned his attention to the English countryside. This article will thus make three interventions into the limited literature on cancer in nineteenth-century England. First, it will suggest that through a process of quantification, tabulation and mapping, cancer came to be constituted as a disease of place and space.^3^ Second, it will show that cancer mapmakers and ‘map thinkers’ were preoccupied with the countryside.^4^ They understood the disease as produced by rural environs, and thus paradoxically correlated to healthful locales rather than areas of urban squalor.^5^ Third, through an interrogation of Haviland’s intentions, this article argues for a reappraisal of the purpose of Victorian medical mapping. Drawing on the work of spatial theorists and geographers, most historians have understood maps in Foucauldian terms as an aspect of governmentality.^6^ Thus, analyses of mapping—particularly in the Victorian context—are often shot through with claims about power, social control, inequality and enforcement.^7^ For example, in his review of the concepts of space and place, geographer Phil Hubbard argues, ‘for some, this twin focus on relationships of power and the politics of representation is the defining characteristic of contemporary cultural geography’.^8^ However, various authors have made efforts to raise the complexity of the historical analysis of maps. For example, Tom Koch suggests that maps are ‘experimental systems’ and that practitioners, like John Snow, deployed cartography in their efforts to argue for a particular aetiological model.^9^ Similarly, this article moves beyond interpreting mapmaking solely through the lens of power and the control of problematic populations and argues instead that cancer prompted mapmakers to attempt to manage the relationship between the rural environment and its human inhabitants.

Today, the notion that certain spaces and places make their residents more susceptible to cancer is familiar. Since the 1960s, anxieties over carcinogenic places, geological landscapes, and environmental pollutants have formed a prominent part of public health discourse.^10^ Moreover, as cancer has become an increasing epidemiological burden on late-twentieth-century society, the suggestion that the disease threatens the health and well-being of national populations is common and uncontroversial. However, we know little about the origins of these ideas because the pre-twentieth-century history of cancer is relatively unstudied.^11^ One reason for this is cancer’s configuration as a ‘pathology of progress’ and its intractable relationship with notions of twentieth-century ‘civilisation’.^12^ Roy Porter and Siddhartha Mukherjee respectively called it ‘the modern disease *par excellence*’, and ‘the quintessential product of modernity’. Both, however, situate that modernity after the Second World War.^13^

Moreover, when cancer’s nineteenth-century history is recounted, it is almost never incorporated into histories of public health.^14^ The literature that does pay attention to the intellectual history of the disease generally places cancer within the context of hospital or laboratory-based investigation.^15^ However, this was just one way of interrogating malignancy. The fact that certain nineteenth-century actors thought of cancer as relevant to the health of spatially configured populations, and constructed and represented this thinking through mapping, has been the subject of only minimal historical scholarship.^16^ In contrast, literature on Victorian public health, sanitary reform, medical statistics and mapping is plentiful.^17^ However, much of this has focused on urban geographies. Haviland looked at rural environments and at cancer on a national or regional scale. Investigating these alternative scales and foci not only adds texture to our understanding of nineteenth-century medical maps, it also tells us something about how cancer was thought to operate. The existence and wide reception of cancer maps suggests that the disease was at least partially integrated within the intellectual landscape of nineteenth-century medicine and public health. Thus, and as this article shows, late-nineteenth-century public health was not just made up of nuisance inspectors, medical officers and the construction of sanitary infrastructures, but can also be thought of as a population-based and spatial way of thinking about disease.

## Cancer, Vital Statistics and Mapping in Victorian England

In 1792, the Middlesex Hospital in London established a ward dedicated to the care of cancer.^18^ From the outset, the hospital staff conceptualised cancer as an integrative category that described a single disease with a range of shared characteristics (manifested by a physical tumour, with the capacity for spread or recurrence, that almost inevitably led to death).^19^ Contemporaneously, the philanthropist Sir Thomas Bernard wrote, ‘In the long train of diseases to which human nature is subject, no one is attended with more hopeless misery than that which is denominated cancer’. He lamented the ‘present insufficiency of medicine’ and confirmed cancer ‘as an incurable disease’.^20^ The hospital board framed the new ward as a solution to the ‘cancer problem’. They were optimistic, ‘If such an Institution be fairly set on foot, it cannot fail of producing beneficial consequences to all descriptions of Persons labouring under this dreadful Malady’.^21^ However, by the mid-century, no solution—either within the hospital or without—had been found. Practitioners continued to be ambivalent over their capacity to accurately identify and successfully treat cancer. Throughout the century, tracts and treatises were replete with lamentations over cancer’s mysterious and incurable status. In 1870, Haviland wrote that cancer was, ‘a disease … which hitherto has baffled all the skill of generation after generation of our professional brethren’.^22^ It was ‘a most painful and loathsome’ malady, one that, ‘kills by inches, and seldom admits of any cure except by the knife, and even that remedy does not always succeed’.^23^ Even at the advent of the twentieth century, the disease was persistently enigmatic. In 1908, *The British Medical Journal* (*BMJ*) wrote,

The greater the amount of ignorance, the profounder the amount of doubt in regard to any subject, the larger is the number of theories, and the more over-whelming the literature on the subject. This is essentially the case with the cancer problem. No doubt many isolated facts in connexion with its histo-pathology have been discovered, and its clinical characteristics have been determined, but the aetiology still remains a mystery.^24^

Moreover, as the century progressed the ‘ignorance’ surrounding cancer’s causes was perceived as increasingly out of step with parallel achievements in understanding, preventing and treating other diseases.^25^

Thus, medical men cast their net wide in search for new diagnostic, investigative and therapeutic methods; and various constituents of the nineteenth-century medical landscape responded to the ‘cancer problem’ in different ways. Men who had learnt their trade in the laboratory attempted to decode the disease with the help of the microscope.^26^ However, while cell theory provided a seductive explanatory mechanism; its influence on clinical practice was more restricted.^27^ From *c*.1840 onwards, case histories of cancer increasingly referred to the use of the microscope. In his 1858 tract on *The Diagnosis of Surgical Cancer*, English ophthalmologist John Zachariah Laurence included descriptions of the ‘minute anatomy’ of all the published cases.^28^ Reflecting on recent medical history in 1864, Irish surgeon Maurice Henry Collis wrote, ‘The combination of microscopic investigation with clinical study has cleared up much that was obscure and unintelligible, and has rendered safe and scientific much that before was empirical in practice. Not only have the means of diagnosis been improved, and treatment rendered more sure, but the results, in a given case, can be predicted with a certainty that we could not have ventured to use a few years ago’.^29^ However, many other practitioners expressed doubts over the value of the instrument in the clinical diagnosis of cancer and particularly over its ability to act alone. John Hughes Bennett, a keen advocate for the microscope in scientific investigation, was also ambivalent about its therapeutic utility, ‘The microscope *alone—*that is, independently of all other kind of observation—can seldom determine in the living subject the presence or absence of Cancer.’^30^ The obstetric physician G. Ernest Herman wrote in 1894, ‘I think the value of the microscope in the clinical diagnosis of cancer has been overestimated. The only use of the microscope is to confirm suspicion aroused by the evidence of the unaided senses of sight and touch.’^31^

Other medical men—those who had matured intellectually and professionally within the context of sanitary reform and the public health movement—tended towards investigating the spatial distribution of cancer, making use of statistics and mapping. In 1838, the *Annual Report of the Registrar-General of Births, Deaths and Marriages in England*, provided a new body of evidence with which statistical questions about cancer and its cause might be answered.^32^ In 1839, the statistician William Farr joined the General Register Office [GRO], and proceeded to tabulate regional and national vital statistics—births, marriages and deaths—for each of the country’s divisions. Thus, the 1840s and 1850s saw the English populace increasingly quantified. This practice derived in part from the development of statistical methods and epidemiology.^33^ The mid-nineteenth-century saw these intellectual movements increasingly institutionalised and professionalised. From the fourth *Annual Report* causes of death were recorded, alongside the person’s sex, age and profession. The causes were divided into ‘Epidemic, Endemic, and Contagious Diseases’, ‘Sporadic Disease of Uncertain or Variable Seat’, ‘Sporadic Diseases of Special Systems and Organs’, and ‘External Causes: Poisoning, Asphyxia, Injuries’. Cancer was categorised as a ‘Sporadic Disease of Uncertain or Variable Seat’.^34^ The GRO also calculated annual mortality by cause. Farr developed various tools to ameliorate the process of gathering and interpreting national data including a standard nosology, standardised death rates, and mathematical models.^35^ Narrative prefaces to each annual report situated individual investigations within a broad chronology, and enabled doctors and public health professionals to comment on yearly shifts in the disease profile of the nation.^36^

As the quantity of data on cancer accumulated, observers began to draw conclusions about the changing incidence of the disease over time. Cancer appeared to be increasing. The *Forty-Second Annual Report*, published in 1879, recorded that among men of all ages cancer was the cause of 4,121 deaths, the same order as diseases like diarrhoea (5,712), whooping cough (5,804), scarlet fever (9,148) and measles (4,678).^37^ Among women of all ages the figures were even more dramatic—8,508 deaths—more than any other disease.^38^ The preface elaborated on these high numbers, and expressed concern over the increased mortality from cancer, which had ‘maintained the increase to which it has been gradually mounting for many years’.^39^ Responses to this supposed increase became increasingly fretful. In 1883, The *BMJ* published an article which lamented that, ‘A cursory examination only is sufficient to divulge that the fell disease [cancer] claims year by year a higher ratio of victims.’^40^ Cancer was growing, hidden, within the social body—mirroring its pathological progress through the textures of internal cells.^41^ Commenters made use of an emotive vocabulary to express their concern, ‘Unhappily … a strict examination of the facts and figures bearing upon it, must lead to the painful and disquieting conviction that cancerous disease is, year by year, becoming more fatal in this country’.^42^ This bleak prognosis—both for individuals afflicted and for the population as a whole—filtered through multiple strata of nineteenth-century society. Concern over the new ‘cancer epidemic’ was not confined to professional discourse—rather evidence for, and debates about, the increase in cancer appeared in a variety of publications, ‘The rapid increase of cases of death by that dread disease cancer’, wrote the *New York Times* in 1902, ‘is exciting attention in Europe as it has here.’^43^

## Dr Alfred Haviland

Dr Alfred Haviland was born in Somerset and trained as a doctor at St Thomas’s Hospital, London.^44^ Various elements of his professional and intellectual background primed Haviland to direct his investigative attentions to cancer and interpret the disease as a problem of space and place. These elements are made clear by his publication history. In 1855 he wrote two tracts, *The Sanitary Regulations of Ancient Rome* and *Climate, Weather, and Disease.*^45^ In 1875, he published his most celebrated work, *The Geographical Distribution of Heart Disease and Dropsy, Cancer in Females & Phthisis in Females, in England and Wales* (reprinted as *The Geographic Distribution of Disease in Great Britain* in 1892).^46^ Followed by *Geology in Relation to Sanitary Science* in 1879, and *Scarborough as Health Resort: Its Physical Geography, Geology, Climate & Vital Statistics* in 1883.^47^ For *The Geographical Distribution* he produced six small and three very large coloured maps (they fold out of the book, covering a desk), showing heart disease, cancer and tuberculosis mortality for England and Wales. In addition, he printed three maps of London showing the distribution of each disease. In the second edition, he created geological and contour maps of the Lake District, overlaying the regional distribution of cancer. *Scarborough as a Health Resort* began with a large-scale (again, fold-out) map of the town, with all its climatological and topographical features carefully engraved.

All of Haviland’s publications reveal a deep commitment to climatological, topographical and geographical determinants of health and disease, further evidenced by lectures on the ‘Geographical Distribution of Diseases’ delivered at St Thomas’s. In *Climate, Weather and Disease* (1855), Haviland wrote, ‘It will be the endeavour of the author in the following pages to present to the student some of the more remarkable facts, that prove the dependence of many diseases, for their origin and continence, on certain meteoric phenomena.’^48^ He waxed lyrical on the value of investigating climate, ‘In studying Climate we study man; for in tracing its effects in all their variety on the human frame and mind, we make ourselves acquainted with his laws, customs, psychical and physical capabilities, vices, virtues, and all that appertains to that protean animal.’^49^ This tendency towards environmental explanations for disease was intimately tied to an Enlightenment, even Hippocratic, tradition of medical geography.^50^ Understanding and remedying ill-health in the eighteenth century was predicated on a long-standing belief that disease was dependent on the place a person lived. Therapeutic advice, for example, was based on the peculiarities of a patient’s environment (as well as their constitutions and habits).^51^ However, such spatial thinking was reinvigorated by the public health movement in the 1830s and 1840s, and then transformed into practical intervention and legislation by reformers such as Edwin Chadwick.^52^

Haviland was fully embedded in the mid-nineteenth-century community of public health practitioners, and invested in sanitary reform as a mechanism to improve the well-being of the population. His books dealt with sanitary reform (*The Sanitary Regulations of Ancient Rome*) and public health (*Scarborough as a Health Resort*, with a preface by J. W. Taylor, the local Medical Officer of Health [MOH]), and he was MOH himself for the combined sanitary authority of Northampton, Leicester, Rutland and Buckinghamshire. In the second edition of *The Geographical Distribution of Diseases in Great Britain*, Haviland reflected on his professional life and the genesis of his interest in geographical and climatic origins of disease, noting the relationship between his experience of public health practice and environmental determinants of ill health. In 1849, he had ‘medical charge of [his] native town, in the West of England, at the time of the direful visitation of Asiatic Cholera epidemic of that year’.^53^ Throughout the pestilence—and desperate for an explanation of cholera’s aetiology—he was ‘constantly taking meteorological observations’ and took note of the ‘relationship between cholera and the wind’.^54^ For Haviland, nature itself was pathological; a commitment he would maintain in his later encounters with cancer.

Haviland’s professional, intellectual and ideological relationship with the public health community also provided him with a technology—maps. In the context of the successive cholera and other disease epidemics throughout the early- to mid-nineteenth century, public health practitioners used mapping to identify particularly pathological areas and justify sanitary reform, typically of urban locales. Edwin Chadwick is credited with bringing medical mapping into the British mainstream. In 1842, he published *The Sanitary Conditions of the Labouring Poor*, a ‘seminal document in the nineteenth-century literature on social welfare’.^55^ In it, Chadwick used basic health and income statistics to map salubrious streets in Leeds, and expressed the variation with colour and shade. This work inspired a proliferation of sanitary maps, largely depicting industrial areas, which were deployed in the investigation of disease in the nineteenth century. Maps of the metropolis and its districts were a regular feature of public health print media, such as John Snow’s famed mid-century diagram of cholera deaths in Soho, London.^56^

Chadwick used maps to make a claim for a miasmatic aetiology of cholera. He argued—along with many others—that the evolving industrial city contained within it specific conditions that predisposed inhabitants to ill health. In this anti-contagionist schema, disease was intimately tied to pathological urban locations—the slum, factory and workhouse. Chadwick and his co-theorists suggested that the environment could both act as a carrier for disease agents (‘ferments’ that could arise *de novo* given favourable conditions), and weaken the body, making it vulnerable to infection.^57^ Maps were to make the complexity of disease easily accessible to the professional gaze, and their necessarily visual structure was not only useful in the actual investigation and practice of public health, but could also be deployed persuasively. In the nineteenth century, maps were increasingly part of material and consumer culture. They featured in schoolrooms, decorated middle-class homes and appeared in the pages of newspapers and periodicals.^58^ As a result, a wider audience was inculcated into the ‘language’ of cartography.^59^ Mapmakers drew on this accessibility to justify their choice in technology.

**Fig. 1:**
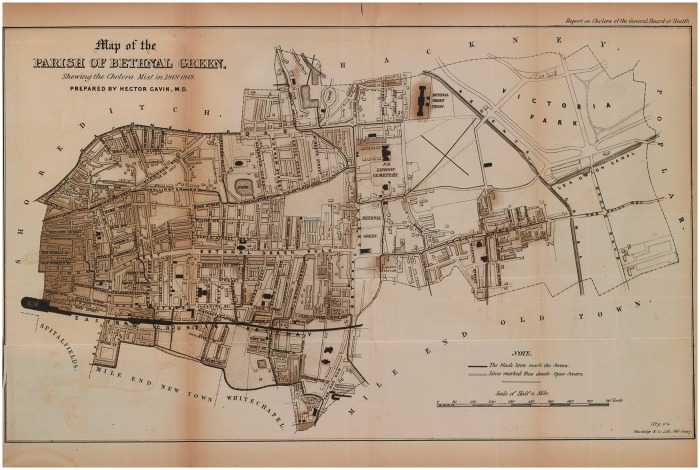
Map of the Parish of Bethnal Green, Shewing the Cholera Mist in 1848–1849, Wellcome Library, London

**Fig. 2:**
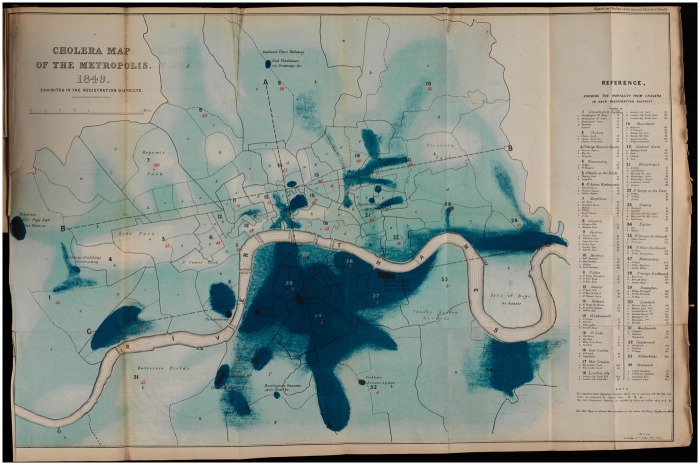
Cholera Map of the Metropolis, 1849, Exhibited in the Registration Districts 1850, Wellcome Library, London

Mapping was designed to make the correlation between disease incidences and different pathological environments visible to the naked eye—to uncover and simplify complex and dynamic relationships between landscape, human behaviour and disease.^60^ These motivations are made clear by the text that supports the various sanitary maps which is replete with visual metaphors. They ‘elucidate’, ‘display’ and ‘reveal’.^61^ Nineteenth-century surgeon and medical historian D’Arcy Power contrasted the relative opacity of numbers and tables with the easily consumable medium of maps, ‘Although the actual numbers are not very imposing in this series of cases, a *glance* at the maps will show the remarkable manner in which the disease is distributed’ [emphasis added].^62^ Debates over cholera’s aetiology also played out in map form. Various diagrams of the disease’s incidence made a case for miasmatic theories, both explicitly and implicitly. For example, the 1849 cholera map of Bethnal Green is labelled as ‘Shewing [sic] the Cholera Mist’, and the shading is evocative of a spreading, dense atmosphere [Figure 1]. A ‘Cholera Map of the Metropolis’ from the same year, similarly uses intensity of colour to call to mind the extension of disease miasma [Figure 2].

The utility of this technology was manifest, and Haviland deployed maps with enthusiasm throughout his long career. However, his ability to make maps and think geographically was itself dependent on the collection and tabulation of vital statistics by the General Registry Office. Haviland was explicit about the debt mapping owed to the quantification of the social body. He was devoted to the GRO’s *Annual Reports—*‘That valuable epitome of knowledge’, in his words—and dedicated his 1855 tract *Climate, Weather and Disease* to the current Officer, George Graham.^63^ The GRO data allowed those interested to assess the distribution of health and ill health across the country, and analyse region by region. Haviland wrote that the GRO reports, ‘rendered the geographical distribution of disease in England and Wales a possibility’.^64^ However, spatial approaches to disease pre-date the GRO. The gathering of data required the division of the nation into political jurisdictions within which health and population information could be adequately collected. Haviland called that process of division ‘mapping’ and in doing so recognised that quantifying the British population was inherently geographical.^65^ The relationship between the numerical method and geography was not one of cause and effect; rather, both emerged from a shared spatial conceptualisation of disease and the population. Public health was, from the outset, geographically configured. Moreover, this geographical configuration arose from the fact that interventions were place-specific. For example, infrastructure (sewage works and slum clearance, for example) necessarily served a specific locality and its impact could not be spread diffusely over regional or national space.

Haviland’s main motivation was to uncover the aetiology of cancer and decode the enigma of its increase. While he thought that his mapping might provide useful information to the practitioner working with cancer patients at the bedside or in the clinic, he also implied that the spatial distribution of the disease across England could address uncertainties about cancer’s intractability and invisibility within the individual body (at least beneath the skin). He argued that understanding the geographical distribution of cancer was ‘a powerful aid in the preventive treatment of many of the grand causes of death’.^66^ More specifically, in 1891, he claimed, ‘one of the functions of the medical geographer is to ascertain where certain diseases prevail, and to indicate those areas on his maps as guides … to the busy medical practitioner who requires to know *at once*, for the sake of the patients who consult him as to where they ought not to reside if they would avoid the diseases they dread, and where are to be found the localities in which there is the greatest chance of escaping them’.^67^ He envisioned his mapping enterprise as relevant to clinical questions.

However, Haviland also went further. He understood cancer to operate on multiple different ‘scales’—from the body all the way through to the nation and repeatedly inscribed the relationship between the individual and social body.^68^ For example, in his rationale for the colour coding of his maps, Haviland indicated that these depictions were intended to represent the human body,

I selected red and blue with the view of aiding the medical memory, the first being typical of red, life-giving arterial blood, the symbol of health and low mortality as indicated by death-rates below the average, while the second represents the colour of effete and used-up venous blood, the emblem of disease and high death-rates, or a mortality above the average.^69^

Just as cancer marked itself on the external landscape of the body—black masses devoid of a healthful flesh—the disease marked itself on the landscape of the nation. Thus, while Haviland’s maps fixed the scale of cancer at the national or sub-national levels, he was also seeking to reveal what was happening at the scale of the human body. For Haviland, therefore, the body (and its scale) can only be understood through reference to the scale of the region or nation. He required environmental representations to make sense of cancer (and chose a rural environment to do so) thus demonstrating that scales are relational and not natural or inherently fixed.

However, cancer often occurred in parts of the living body invisible to the clinical gaze. Medical men were aware that malignancy could navigate its way through the internal textures of the body, without necessarily manifesting external signs. Cancer was repeatedly framed as an unknown or mysterious disease, and primary lesions and metastases alike were often undetectable until after a patient died. The medical philosopher, Elisha Bartlett, spoke at length on the obscurity of cancer, making use of a variety of visual metaphors,

Almost all diseases are occasionally so impressed and modified, by inappreciable or unknown influences, that their usual diagnostic signs are wanting, or very much obscured,—the diseases being *latent*, as it is called. Cancerous disorganisation of the stomach, in some instances, gives no indication of its existence, insufficiently distinct to render its detection possible, during life, even by the most competent and careful observers.^70^

Putting cancer on the macro level of maps thus gave it a visibility that it lacked at the micro level of individual examination. Haviland wrote in 1875, ‘Perchance *some light might be thrown upon* the aetiology of that fatal class of malignant diseases, registered as causes of death under the term cancer … were to be treated on the same geographic principles as had been demonstrated in the cases of phthisis and heart disease’.^71^

Moreover, Bartlett’s use of ‘disorganisation’ to describe cancer was both common and metaphorical.^72^ Alluding to the anxieties provoked by the diseased state, surgeon Walter Hayle Walshe wrote, ‘the fact of a sanies of fetid odor and peculiarly acrid qualities being more or less abundantly thrown out by the disorganised surface’.^73^ The surface was ‘disorganised’—no longer in its proper order, no longer aligning with what we expect and can predict. For Haviland, mapping could reveal the ‘obscured’ cancer, organise the ‘cancerous disorganisation’, and detect what clinical observation had thus far failed to observe.^74^ The inability to identify and treat latent cancers was connected intrinsically to the ‘disorganisation’ noted above, which not only suggests a metaphorical relationship between the practices of public health mapping and the practicalities of detecting cancer in the individual, but also a more fundamental way of thinking with scale. Just as the organisation of public health knowledge could constitute a ‘treatment’ for the social body, the lessons of public health seemed relevant, and even useful, for practitioners confronting cancer in the individual body.

Haviland outlined his general methodology for understanding the cause of cancer in an ‘Abstract of Lectures on the Geographical Distribution of Diseases in England and Wales’, delivered at St Thomas’s Hospital, London, and published in the *BMJ* in 1870:

The mapping of England and Wales in 11 divisions, 53 counties, and 625 union districts, affords the means of analysing the distribution of heart-disease or of any other cause of death. By this threefold division we are enabled to sift the facts through three gauges of different degrees of fineness. In the first space, we see what proportion the death-rate from a cause of death bears to the population in each of the eleven divisions; we colour blue or red those divisions which are above or below the average, and then study this gross distribution carefully. The next process is to colour the counties in the same way, and observe where the distribution at all coincides with that of the divisions. … Having done this, we again review our work, and calculate the effect of each of the many causes surrounding us in the production of the distribution, which our coloured map reveals.^75^

**Fig. 3:**
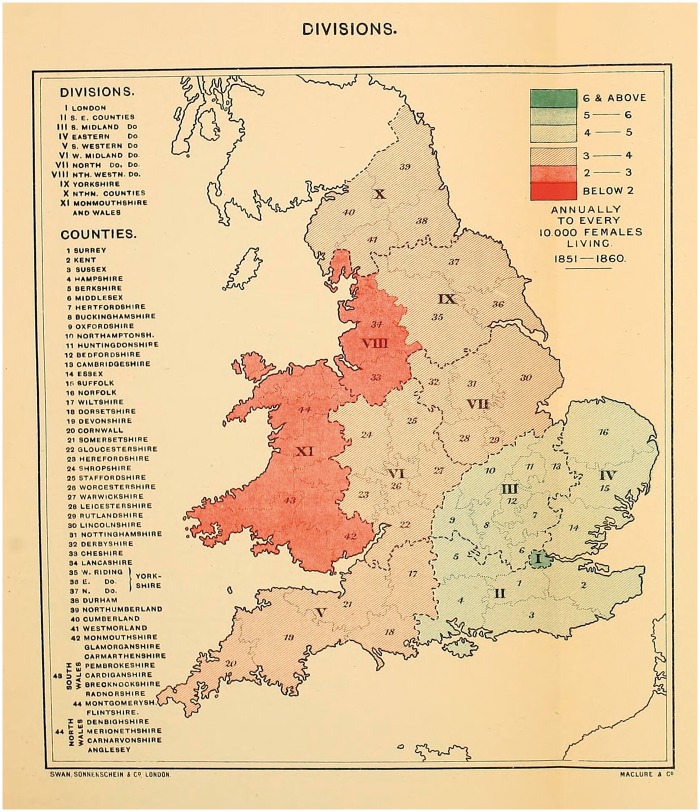
Alfred Haviland, The Geographical Distribution of Cancer (Females), Divisions, 1851–1860, Wellcome Library, London

**Fig. 4:**
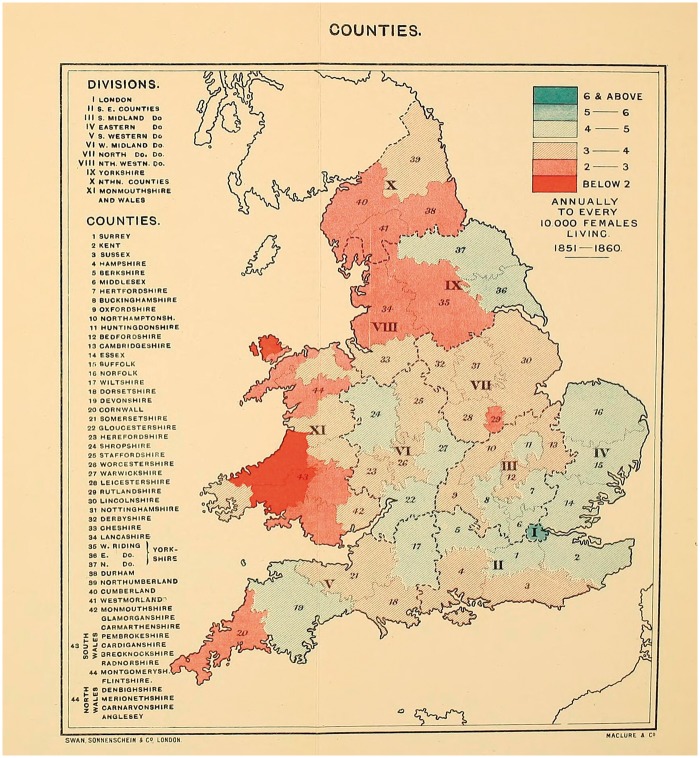
Alfred Haviland, The Geographical Distribution of Cancer (Females), Counties, 1851–1860, Wellcome Library, London

Here, Haviland claimed that the distribution of disease—arranged visually—allowed the observer to explain any variations in incidence. ‘To ascertain the geographical distribution of a disease,’ he suggested, ‘is the first step towards a knowledge of its natural history.’^76^ Rather than, say, examination of an individual body or its component parts. He used mapping ‘to discover where diseases prevail, and were they do not thrive’, and ‘to search for, in those localities, the causes of prevalence, or absences, or scarcity, whether they reside in their local airs of waters, or are due to general or local climates, geological structure, physical configuration, or social surroundings’.^77^ Using GRO data for 1851–60, he mapped out female cancer incidence in England and Wales [Figures 3 and 4].^78^ Havilland’s choice of colour is significant. Chloropleth mapping was first used in France in 1826, and geographers have commented on the nineteenth-century transformation of colour from a nonessential decorative supplement into an integral and functional element of design, indispensable to the ‘cartographic objective’.^79^ As argued above, his colours were meaningful, ‘The lowest mortality is indicated by the darkest red and the highest by the darkest blue.’^80^ In his maps of ‘The Geographical Distribution of Cancer Females, 1851–1860’, the relatively cancer-free arterial blood drains from west to east, with London a malignant blue blot.

The map of the ‘Divisions’ [Figure 3], provided insufficient detail and so Haviland zoomed in on the ‘Counties’ [Figure 4]. If the former suggested an east–west contrast in cancer incidence, the latter presented a more complex picture. His maps of heart disease had been clear in their implications—it was ‘more fatal in the unventilated valley-system of England and Wales than in the open areas freely-exposed to the prevailing winds and sunshine’.^81^ This relationship aligned neatly with commonly-held nineteenth-century assumptions that sunshine and free-flowing, dry air had therapeutic (or preventative) health benefits. Cancer, while no doubt associated with the landscape, was harder to explain.

For Haviland, ‘the high mortality groups’ could be ‘seen to skirt the lower courses of fully-formed rivers that seasonally flood the riparian districts’. Indeed, the intense blue of London could be explained by its straddling of the Thames, ‘The Thames Basin has long been known as one of the great cancer fields of England and Wales.’^82^ Broadly speaking, Haviland concluded that cancer was more fatal among women in ‘clayey flooded areas than on elevated calcerous soil’.^83^ He had a low opinion of clay:

In the history of diseases clays are connected with the most deadly scourges to which the human race has been subjected, such as those that have arisen in our own times from vegetable decomposition after floods—as in the intense of cholera from the alluvial clays forming the delta of the Ganges, and in the long list of malarial fevers all over the world which have had their origin in the deltas of rivers and inland marshes, characterised by alluvial clays saturated with the products of the decomposed and decomposing vegetation, that had first been flooded, then killed, and lastly, left to rot in the sun.^84^

In contrast, his commentary on limestone was distinctly positive: ‘Limestones have no such an appalling record. We know of no epidemic sweeping over the world, either air-borne or man-borne, that could be traced to a limestone nidus; on the contrary … they are associated with the earliest dawn of life.’^85^ Rivers, flowing over clay soil, were the cause of cancer. ‘In the counties having a high mortality from cancer we find that the tributaries of the large rivers flow from soft marly or other easily disintegrated rocks into sheltered valleys, through which the main rivers flow.’^86^

While Haviland does not cite Max von Pettenkofer (1818–1901), professor of hygiene in Germany, his work shares much with the latter’s concept of *Bodentheorie* (soil theory). In the 1860s von Pettenkofer claimed that soil pollution was the principal cause of epidemics, and specifically cholera.^87^ This connection between soil and disease was widespread, and reflected the persistence of localist-miasmatic aetiologies into the late nineteenth century. Men such as Charles Murchison and Alfred Smee, as well as von Pettenkofer, argued that disease-causing germs had to undergo a period of incubation in the soil to become infective.^88^ It was thus consistent to think of cancer as causally related to soil quality and Haviland was well-situated within an established intellectual context. However, Haviland was vague about the precise relationship between rocks, soil, rivers and cancer. While he referenced the possibility of a cancer ‘germ’, he stopped short of setting out any clear aetiological model.

Although Haviland was the most prolific cancer mapmaker, he was not alone in conceptualising the disease as a problem of geography or climate. Indeed, environmental conceptualisations of cancer appeared before Haviland’s mapmaking, and persisted well into the twentieth century. Charles H. Moore, surgeon to the Middlesex Hospital’s Cancer Ward in the middle of the nineteenth century, published a book in 1865 that was full of spatial thinking. Working to uncover the cause of cancer he suggested, ‘Somewhere, among the personal, social, industrial, traumatic, or geographical conditions of the patient, in the *debris* of foregoing disease, or in his ancestral entail, the case of Cancer surely lies within reach of an adequate search.’^89^ Devoting a chapter to the ‘geographical conditions’, he posited that, ‘if a disease common to all the human race is yet unequally distributed, some cause influencing its prevalence or its rarity might be disclosed by an examination of the circumstances’.^90^ He suggested that there must be meaning in the unequal distribution of cancer in Britain, ‘Can we find in the distribution of Cancer among large masses of people, any rule which would connect its rarity or frequency with the general conditions of their life?’^91^ He connected cancer to broader understandings of disease and locale: ‘It is notorious, that very different states of general healthiness exist in large divisions of the community.’^92^ He, like Haviland, relied on the governmental statistics and laid out his rationale for taking death-rate as a reliable stand-in for incidence: ‘In the instance of a malady so fatal as Cancer, the death-rate only too accurately represents its numerical prevalence among the people, the Government returns are suitable for the inquiry before us.’^93^ From that data he concludes, ‘If the country be divided by a line from Bristol to Peterborough (between South-Western and West Midland in the Table), the mortality from Cancer in the five southern divisions is considerably in excess of that on the north of the line. Its greatest prevalence, according to the records, is in London and the counties south of it.’^94^ Moore made limited effort to explain this unequal distribution, but in setting out his justification for the utility of geographical investigations into cancer he laid the groundwork for subsequent efforts to turn such thinking into mapmaking.^95^

Nor was Haviland's the last word on cancer mapping. Studies into the spatial distribution of cancer proliferated in the decades surrounding 1900, and many were regional in focus. The Collective Investigation Committee of the British Medical Association published a map of the distribution of cancer across the British Isles in 1889.^96^ The Committee circulated an ‘inquiry paper’ to every registered medical practitioner in the United Kingdom, which asked, ‘Are the following diseases, or any of them, common in your district; that is, would a medical man in average practice in it be likely to meet with, on the average, a case a year?’ They inquired about rickets, acute rheumatism, chorea, urinary calculus and cancer. As in Haviland’s maps, places in which the disease was ‘common’ were coloured blue, and those where it was ‘uncommon’ were marked with red. More than 3,000 completed papers were returned, and eight maps were produced from the information contained: one of England and Wales as a whole, one of Scotland, one of Ireland, one of the Orkney and Shetlands Islands, one of the Channel Islands, one of Manchester, one of Edinburgh, and one of Greater London. The disease appeared to be fairly evenly distributed, and any clustering in the major cities was explained by the accompanying report in the *BMJ* as a result of the density of medical institutions in these urban places.^97^ Cancer seemed to be particularly common in the Orkney and Shetlands Islands, and in the Channel Islands. In London, the report noted how cancer ‘tended to collect in the flat lands adjacent to the river’, and they referenced Haviland’s riverine thesis.^98^

The Collective Investigation Committee’s maps gestured towards cancer as environmentally determined and more common in rural places. These associations were also picked up by slightly later commentaries, that also posited a relationship between cancer and civilisation. In 1908, the *BMJ* published an article on the correlation between light and cancer incidence. Its author argued that the ‘increase of cancer within the last seventy-five years is perhaps due to diminished protection from light and increased exposure to illumination’. But he also made a geographical argument: cancer was caused by sunlight. ‘Narrow streets and dark houses’, for example, had once been a protection, but now ‘suburban life has largely replaced that of the city’.^99^ In 1909 the *BMJ* published an inquiry into cancer in New Zealand which was principally designed to explain ‘the persistent increase in the percentage of deaths from cancer’. The authors claimed, ‘It will be noticed that many of the cases occurred within a particular area’, before describing the landscape in detail, ‘Between a large snow-fed river and a smaller stream lies a flat tract of country extending in length for about ten miles to the foot of a hill … the base of which was formerly, and is still to some extent, covered with dense native bush.’ They note a change in the flora: ‘Originally this land was covered with tussock—the native grass—and in the more swampy parts with native flax and “nigger heads”. … The nature of this country has now entirely changed, the land being now subdivided into farms, and ploughed; cereals and turnips being chiefly grown.’ They suggested this shift towards agriculture as one potential cause for the increase in incidence of cancer, but provided a range of other environmental determinants as well, from rainfall to the existence of a sluggish creek.^100^ Finally, the article concluded with the authors giving their support to Haviland’s riverine thesis.

Haviland proved a point of reference for many of these analyses (which generally faded into obscurity), and for subsequent general reflections on the geographical distribution of cancer. For example, in 1898 the *BMJ* referred to the ‘well-known views of Mr Haviland’.^101^ In 1899 Cambridge doctor E. Lloyd Jones published an article entitled, ‘The Topographical Distribution of Cancer’, in which he ‘sought to determine in what manner cancer is distributed in the borough of Cambridge and in the surrounding county’.^102^ He referenced Haviland repeatedly, claiming that ‘most observers agree with Haviland that limestone and chalky districts are comparatively free from cancer’.^103^ Haviland also appeared in a 1903 *BMJ* article on cancer mortality: ‘The connexion of the disease with geological formation as shown by Haviland in his cancer map of England and Wales.’^104^ In 1904, Alexander Urquhart wrote in the *BMJ*, ‘The south-eastern division of England has long been regarded as showing a high death-rate from cancer, and the Thames valley in particular has had this unenviable reputation.’^105^ He named Haviland and applied new statistics to an old problem, which seemed to, ‘justify the conclusion that the Thames valley is still associated with a relatively high mortality from cancer’.^106^ Haviland’s theories and data were being talked about in the medical and public health press well into the 1960s.^107^ He was known beyond Great Britain and was referenced favourably in a French medical thesis from 1897.^108^ He was given a long obituary in the *BMJ* and his work was reviewed rapturously in various periodicals. The *Medical Times and Gazette* wrote about *The Geographic Distribution of Disease*: ‘It is a national work, and hence the author has a right to expect to find upon the list of his subscribers at least every sanitary board, not only in England and Wales, but wherever the English language is read.’^109^*The British and Foreign Medico-Chirurgical Review* added, ‘The undertaking is novel, an honour to British Medicine, and calculated to promote the pursuit of a department of pathology hitherto greatly neglected.’^110^ Thus, Haviland’s ideas about the pathological potential of Britain’s natural environment had purchase.

## Cancer and the Countryside

**Fig. 5:**
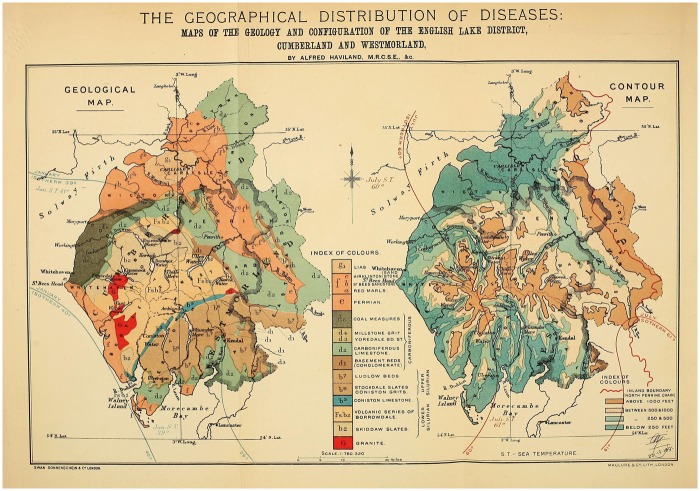
Alfred Haviland, Maps of the Geology and Configuration of the English Lake District, Cumberland and Westmorland, Wellcome Library, London

**Fig. 6:**
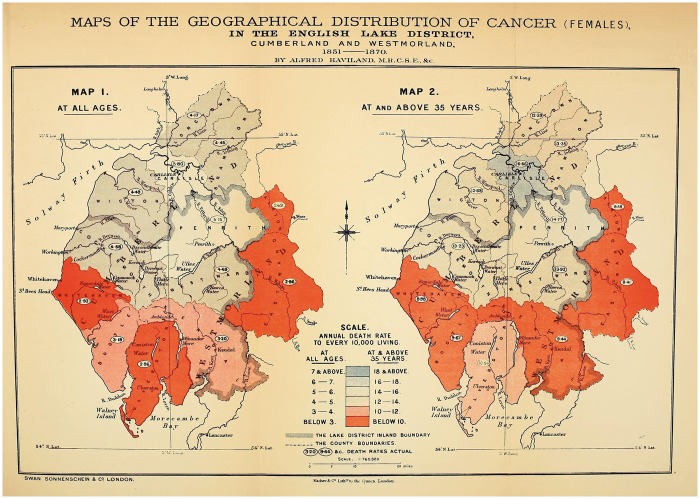
Alfred Haviland, Maps of the Geographical Distribution of Cancer (Females), In the English Lake District, Cumberland and Westmorland, 1851–1870, Wellcome Library, London

Despite Haviland’s obvious dependence on public health practices and aetiological theories, his mapping of cancer differed in a crucial aspect from the sanitary maps of other epidemiologists. Rather than conceptualising cancer as a disease of towns and cities, Haviland framed it as a disease of rural environs. Much of late-eighteenth- and nineteenth-century rhetoric was devoted to pathologising the city, as Noah Webster wrote: ‘Why should cities be erected if they are only to be the tombs of men?’^111^ In contrast, Haviland mapped the agrarian and riverine Lake District. He plotted four maps: a geological map of the theoretical rocks and soil distribution [Figure 5, left-hand side], a contour map [Figure 5, right-hand side], a map of cancer at all ages [Figure 6, left-hand side] and a map of cancer at over 36 years [Figure 6, right-hand side].^112^ He then correlated areas of high mortality and areas of low mortality with the geological substrata and the topographical features: ‘I studied the registration district-map of England side by side with an early impression of Greenough’s splendid physical and geological map of England and Wales.’^113^

Applied to the Lake District, Haviland’s riverine thesis showed areas of ‘blue colour, indicating a high mortality’, marking out, ‘the districts through which the fully formed river *Eden* has its course, and the riparian lands of which it seasonally floods’. In Victorian literary tropes, the Lake District and the Thames Valley were usually associated with health, affluence, images of bucolic England and British national identity. In reconceptualising them as potentially diseased places, Haviland marked himself out as distinctly counter-cultural. An 1883 *Spectator* article on a defeated plan for a new railway that would cut across the region waxed lyrical on the rural beauty of the Lakes, ‘We turn and pass down the vale, by the side of Ennerdale Water. … One thing strikes us at once. The singular loveliness of the wild strip of land between lake and mountain-wall.’^114^ It goes on to suggest that the Lakes were distinctly healthful and beneficial to England’s ‘true wealth’: ‘Parliament has been wise in remembering that England’s true wealth lies not in her mineral supply, so much as in her supply of healthy souls in healthy bodies. Parliament does not forget that the work of the world demands that there shall be rest-spaces for the weary workers.’^115^ The Lakes provided an essential reprieve from the traumas of industrial labour. They were a favourite destination for tourists and visiting dignitaries alike in the nineteenth century, with the King of Saxony including The Lakes in his tour of Northern English attractions in 1844.^116^ In lacing the Lake District with pathological potential, Haviland was remapping understandings of the Victorian landscape.^117^

These various spatial configurations of cancer also tell us something more diffuse about how the disease’s aetiology was conceptualised in the nineteenth century. Haviland and others all understood cancer as produced by rural places. *The Fifth Annual Report of the Registrar-General* recorded that ‘more deaths were referred to scrofula and cancer in the country than in the town districts.’^118^ Haviland writes about the cancer-causing properties of the Avon, the Severn and the Wye rivers, he traces the Trent and the ‘great Yorkshire rivers’, and locates pathological potential in the ‘high ridges of the Northumbrian and Cumbrian hills’. There was a paradox in this correlation between countryside and cancer: if the bucolic was also disease-causing, how did that sit with contemporary conceptualisations of the city as marked by industrial overcrowding and the attendant poverty and poor health? Nonetheless, this fit with broader understandings of cancer that existed in the second half of the nineteenth century. Global geographies of the disease plotted populations on a gradient—at one end sub-Saharan African colonies, at the other Anglo-Saxon or Teutonic races. Doctor Hugh P. Dunn wrote, ‘observation has shown that cancer has a certain geographical distribution. It prevails extensively in some parts of the globe, and is scarcely known in others’.^119^ This mapping was marshalled as evidence for cancer as a ‘disease of civilisation’.^120^ Not only was the disease on the increase, the epidemic was confined to nations that were understood as biologically, culturally and economically superior. And, by extension, in the domestic context cancer was flourishing in the rural idyll.

In this way, cancer came to be reconceptualised as a disease of health and affluence. This was both explicit and implicit in the writings of medical men and their lay counterparts. Dunn suggested, ‘cancer is said to abound in the healthiest districts and amongst the people who are most robust’.^121^ This claim was supported by close statistical analysis, undertaken by the Scottish statistician and president of the Royal Statistical Society (1947–49) David Heron. He wrote in 1906, ‘The conditions of prosperity and culture which lead to a low birth-rate also conduce to a high cancer death-rate. In other words, cancer cannot, like phthisis, be taken as a measure of that unhealthy environment with which a high birth-rate seems to be associated.’^122^ Here, Heron inverted contemporary speculation that falling birth rates were the result of national decline and degeneration.^123^ For Dunn and Heron the relationship between cancer and civilisation was *unlike* the conceptualisation of various diseases of poverty such as cholera, rickets and typhoid. Cancer may have been a pathology of progress, but it was not caused by industrialisation and its well-known pathological corollaries: filth, overcrowding, lack of sunlight and moral depravity.^124^

However, Haviland’s mapping of cancer in rural environs also reveals something subtler in his understanding of mapping and its role in public health. His mapmaking—in so many ways familiar to the standard narrative of maps as the insidious and modernist tools of state control over deviant *populations*—here seems to veer off in a different direction, used to interrogate *nature*. While the Thames Valley was coloured deep blue—indicative of high cancer mortality—the metropolis was not the cause of elevated incidence. Instead, it was the water, soil, rock—the ‘natural’ environment. While cholera and cancer both might be dependent on their environments, they were produced by very different places. This not only shows us that public health in the nineteenth century was concerned with non-urban places, but that cancer was perceived as fundamentally different to the epidemic diseases of urban poverty. Mapping was, therefore, not simply an expression of Victorian anxieties about the new industrial town, but could equally be applied to districts representative of health and well-being.

Chadwick and his allies were explicit in their understanding of the urban environment as pathological; however, historians have tended to read against the grain and interpret sanitary reform and the mapmaking that accompanied it as an attempt to organise *people* as well as, or instead of, *spaces*. Haviland’s interest in rural places might be understood as a way to organise the landscape into submission, but at heart he was more interested in the management of the relationship between people and places and an implicit return to Hippocratic ideas. As argued above, Haviland thought of his mapmaking as usefully preventative, suggesting a way for humankind to ‘manage’, through mapping, their relationship with nature into something healthy. The disease-causing properties of certain places might not be alterable, but they could at least be avoided.

## Conclusion

This article has demonstrated how mapping reveals the extent of cancer’s integration within the medical landscape of the nineteenth century. The theoretical arsenal applied to decoding its aetiology was dependent on new statistical approaches to the health and well-being of the population. Haviland—a public health practitioner—applied the same technologies to cancer that had been used on cholera and other epidemic diseases. The mapping of cancer was in dialogue with an environmental conceptualisation of disease causation, and cancer maps were frequently deployed to argue for a climatological or geographical determinant of malignancy. Moreover, between *c*.1860 and 1914 cancer was constructed as a disease of place, in tandem with its transition from a disease of individual tragedy to a public health problem. Beyond literal formulations of cancer as environmentally determined and its location on material maps, the conceptualisation of spatial malignancy reflected the metaphorical language used to describe the pathology of cancer. Pathologist Frank Bushnell and biologist F. Cavers wrote in 1904, ‘Observations are being made with a view to mapping out the topography of cancer cells.’^125^ Cancer was a disease *located* somewhere within the bodily textures—it could ‘travel’ from organ to organ and had its own internal geography.^126^

**Fig. 7:**
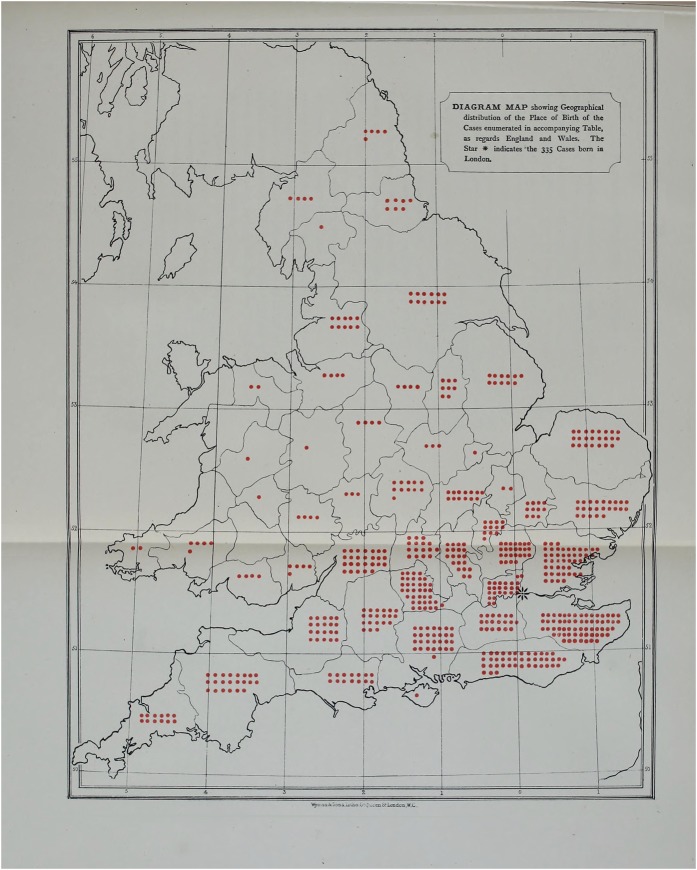
T. W. Nunn, Diagram Map showing Geographical Distribution of Breast Cancer Cases at the Middlesex Hospital, 1882, Wellcome Library, London

Close metaphorical links were forged between cancer of the corporeal body and cancer within the national landscape. Much like its movement through the corporeal body, cancer’s presence in the *body politic* was diffuse. The disease travelled along tributaries (along lymphatic channels or systems of rivers and streams), and infected distant parts. Moreover, cancer featured on maps of the whole country, rather than just cities or towns [Figure 7].^127^ Cancer was seen not only to affect (rural) parts of the population largely otherwise neglected by public health practitioners and historians alike, but it was understood as a disease of relevance to the entire nation state. In this period, therefore, cancer came to be reconceptualised as a malady that affected the population—people in aggregate—rather than primarily a disease of the individual clinical interaction. This was only possible after cancer had been—quite literally—placed on the national map and integrated with the collection of population statistics. In other words, mapping made cancer comprehensible within the framework of a nationally oriented public health.

Haviland, therefore, offers an unexpected point of origin in the development of the twentieth-century notion of cancer as a public health problem. While his aetiological frameworks might be alien, the relationship he posits between cancer and place is familiar to us. Moreover, while I may have argued for an integration of cancer into the history of Victorian public health, I contend that this integration will not be easy or simplistic. Haviland’s chosen scales and foci reveal something not just about cancer itself, but about the larger context of mapping and modernity. In some ways, the curious case of Haviland aligns with what we already know about the rationales for public health in the nineteenth century. He can be read as committed to sanitary reform and statistical methodologies, and as working to make visible an obscure and threatening disease. However, unlike sanitary mappers, he was not preoccupied by the threat of urban ‘civilisation’ and industrial overcrowding. Instead, cancer maps overwhelmingly represented rural places. Thus, Haviland’s work subtly undermines the tendency on the part of some historians and historical geographers to only read power and social control into their analyses of maps, sanitary reform and public health in the nineteenth century.

Haviland’s maps indicate that we need to take nineteenth-century attitudes to rural environments and their public health implications seriously, however, they also allow for a reconsideration of Victorian approaches to urban social and medical pathology. While his choice of place cuts across our expectations, Haviland’s motivations are also left unclear. Unlike Chadwick *et al*., he made few policy recommendations. He seemed resigned to the inevitability of cancer: it seeped out of soil and ran through rivers. Haviland thought of his mapmaking as usefully preventative, suggesting a way for humankind to organise, through mapping, their relationship with nature into something healthy. His maps visualised a way of living in pathological places. The disease-causing properties of certain places might not be alterable—mountains, rivers and streams cannot be moved—but they could at least be avoided.

## Footnotes

1 Alfred Haviland, *The Geographical Distribution of Heart Diseases and Dropsy, Cancer in Females and Phthisis in Females, in England and Wales* (London: Elder Smith, 1875).

2 Haviland was also indebted to a rich nineteenth-century culture of medical geography. However, the most famous of these publications, August Hirsch’s *Handbuch der historisch-geographischen Pathologie*, makes only minimal reference to cancer. August Hirsch, *Handbook of Geographical and Historical Pathology*, trans. Charles Creighton, 2 vols (London: New Sydenham Society, 1883–86), II, 96, 599. See, Nicolaas A. Rupke (ed.), *Medical Geography in Historical Perspective* (London: The Wellcome Trust Centre for the History of Medicine at UCL, 2000).

3 While the two terms are often used synonymously, human geographers have sought to differentiate between the related but distinct concepts of ‘social space’ and ‘lived-in place’. Here, I tend towards using ‘place’ because while mapmaking purported to produce abstract ‘space’, the process by which men like Alfred Haviland mapped cancer incidence populated certain spaces with meaning—thereby turning them into ‘places’. David Atkinson, Peter Jackson, David Sibley and Neil Washbourn, eds, *Cultural Geography: A Critical Dictionary of Key Concepts* (London: I.B. Tauris, 2005), 41. In other words, cancer mapping transformed the abstract ‘space’ of the British landscape into a meaningful, bounded ‘place’ with a range of implications. See, Courtney J. Campbell, ‘Space, Place and Scale: Human Geography and Spatial History in Past and Present’, *Past and Present*, 2016, 1–23.

4 ‘I have borrowed the term ‘map thinkers’ from Tom Koch. Tom Koch, *Disease Maps: Epidemics on the Ground* (Chicago: The University of Chicago Press, 2011), 149.

5 Keir Waddington argues that while historians have recently attended to what he calls the ‘local dynamics of public health’, these existing studies ‘tend to discount the rural’. He has also shown how the ideological meanings attached to the Victorian landscape were ‘plural’, ranging from romance, morality and health, to a ‘growing disquiet’ about rural overcrowding, poor quality housing and inadequate sanitation. Keir Waddington, ‘“In a Country Every Way by Nature Favourable to Health”: Landscape and Public Health in Victorian Rural Wales’, *Canadian Bulletin of Medical History*, 2014, 32, 183–4. See also, Keir Waddington, ‘“I should have thought that Wales was a wet part of the world”: Drought, Rural Communities and Public Health, 1870–1914’, *Social History of Medicine*, 2017, 30, 590–611.

6 Patrick Joyce argues, ‘the modern map is essential to power and to the practices of governance’. Patrick Joyce, *The Rule of Freedom* (London: Verso Books, 2003), 36.

7 J. B. Harley argued that cartography, and the cartographic vocabulary, ‘embodies a system of social inequality. The distinctions of class and power are engineered and legitimated in the map by means of cartographic signs’. J. B. Harley, ‘Deconstructing the Map’, *Cartographia*, 1989, 26, 1–20, 6.

8 Phil Hubbard, ‘Space/Place’, Atkinson *et al*., *Cultural Geography*, 41–8, 46.

9 Koch, *Disease Maps: Epidemics on the Ground*, 13. Susan Schulten also argues that in the nineteenth century, ‘maps became one of the most promising ways to hypothesize about contagion and transmission’. Susan Schulten, *Mapping the Nation: History and Cartography in Nineteenth-Century America* (Chicago: Chicago University Press, 2012), 90.

10 There are various late-twentieth-century examples of this, including the carcinogenic impacts of nuclear power stations, radon-emitting granite in Cornwall, and the April 1990 cover of the *Journal of the Louisiana State Medical Society* (1990, issue 4) suggested a so-called ‘Cancer Alley’ along the shores of the Mississippi between Baton Rouge and New Orleans.

11 There are a few early-modern and eighteenth-century studies of cancer: Marjo Kaartinen, *Breast Cancer in the Eighteenth Century* (London: Pickering and Chatto, 2013), and Alanna Skuse, *Constructions of Cancer in Early Modern England: Ravenous Natures* (Basingstoke: Palgrave Macmillan, 2015). Some historians have addressed cancer in the nineteenth century. See, Carsten Timmermann, *A History of Lung Cancer* (Basingstoke: Palgrave Macmillan, 2013). For accounts of cancer in nineteenth-century America see, Robert A. Aronowitz, *Unnatural History: Breast Cancer and American Society* (Cambridge, MA: Harvard University Press, 2007); and James T. Patterson, *The Dread Disease: Cancer and Modern American Culture* (Cambridge MA: Harvard University Press, 1989). Scholars of gender and medicine have paid particular attention to breast cancers and gynaecological malignancies. See, Tammy Duerden Comeau, ‘Gender Ideology and Disease Theory: Classifying Cancer in Nineteenth Century Britain’, *Journal of Historical Sociology*, 2007, 20, 158–81; Ilana Lowy, ‘“Because of their Praiseworthy Modesty, They Consult Too Late”: Regime of Hope and Cancer of the Womb, 1800–1910’, *Bulletin of the History of Medicine*, 2011, 85, 356–83; and, Ornella Moscucci, *Gender and Cancer, 1860**–**1948* (London: Palgrave Macmillan, 2016).

12 Cancer’s particular association with the twentieth century refracts through academic and lay discourse, including in epidemiological and public health literature: Abdel Omran, ‘The Epidemiological Transition: A Theory of the Epidemiology of Population Change’, *The Milbank Quarterly*, 1971, 83, 731–57.

13 ‘The story of cancer as a distinctively modern … entity’, writes Steven Shapin in his New Yorker review of Siddhartha Mukherjee’s book *The Emperor of All Maladies: A Biography of Cancer*, ‘starts … in 1940’; Steven Shapin, ‘Cancer World: The Making of a Modern Disease’, *New Yorker*, 8 November 2010.

14 For example, *An Atlas of Victorian Mortality* does not have a separate chapter on cancer. Robert Woods and Nicola Shelton, *An Atlas of Victorian Mortality* (Liverpool: Liverpool University Press, 1997). However, the exception is Ornella Moscucci in her article, ‘Gender and Cancer in Britain, 1860–1910: The Emergence of Cancer as a Public Health Concern’, *American Journal of Public Health*, 2005, 95, 1312–21. This is particularly surprising considering that Arthur Newsholme—a key character in the story of Victorian public health—turned his attention to cancer later in his career. George King and Arthur Newsholme, ‘On the Alleged Increase of Cancer’, *Proceedings of the Royal Society of London*, 1893, 54.

15 See, L. J. Rather, *The Genesis of Cancer: A Study in the History of Ideas* (Baltimore: Johns Hopkins University Press, 1978).

16 One of the only scholars to contend with cancer mapping in the nineteenth century is Tom Koch, *Cartographies of Disease: Maps, Mapping and Medicine* (Redlands, CA: ESRI Press: 2005).

17 See, Edward Higgs, *Life, Death and Statistics: Civil Registration, Censuses and the Work of the General Register Office, 1836**–**1952* (Hatfield: Local Population Studies, 2004); and, Pamela K. Gilbert, ‘The Victorian Social Body and Urban Cartography’, in Pamela K. Gilbert, ed., *Imagined Londons* (Albany: State University of New York Press: 2007).

18 See, Bruce Schoenberg, ‘A Program for the Conquest of Cancer: 1802’, *Journal of the History of Medicine and Allied Sciences*, 1975, XXX, 3–22; and Samuel Whitbread, ‘Address: to the Governors of the Middlesex Hospital’, in Samuel Young, *Minutes of Cases of Cancer and Cancerous Tendencies Successfully Treated by Mr. Samuel Young* (London: Printed for E. Cox and Son and J. Ridgway by J. M. Creery, 1815).

19 In this article, I address cancer as a collective term. Victorian practitioners distinguished between different types—breast, lung, and skin, for example—and there was considerable debate at the ‘margins’ of the category with particular disagreement over the status of maladies that shared experiential characteristics like lupus, scrofula, and consumption. However, I suggest that the disease moved through medicine and society as an integrated unit; that it is reasonable to speak of a broadly shared ‘cancer experience’; and that the disease had an undifferentiated social significance.

20 Thomas Bernard, ‘Extract from an account of the Institution for Investigating the Nature and Cure of Cancer’, *Reports of the Society for Bettering the Condition and Increasing the Comforts of the Poor*, 1802, 3, 355.

21 UCLH Archive, London, ‘Weekly Board Meeting’, Middlesex Hospital Minutes, 10 January 1792.

22 Haviland, ‘Abstracts of Lectures on the Geographical Distribution of Disease in England and Wales’, II, 574.

23 William Buchan, *Domestic Medicine; or A Treatise on The Prevention and Cure of Diseases, by Regiment and Simple Medicines* (London: Milner and Sowerby, *c*.1870), 320.

24 ‘Hypothesis and Treatment in Relation to Cancer’, *BMJ*, 1908, 2, 1509–10, 1509.

25 The doctor and writer Hutchinson Woods wrote, ‘We have banished the plague, drawn the teeth of small-pox, riddled the armour of diphtheria, and robbed consumption of half its terrors … our bills of mortality show a marked diminution in the fatality of every important disease which afflicts humanity’, Hutchinson Woods, ‘The Cancer Problem: Treason in the Republic of the Body’, *The Contemporary Review*, 1899, 76, 105–17, 105.

26 L. S. Jacyna has argued that cell theory was readily accepted in British medical circles. Most mid-century practitioners incorporated versions of Johannes Muller’s and Rudolf Virchow’s tumour cell theory into their understandings of cancer’s aetiology. On the reception of Virchow in Britain, see L. S. Jacyna, ‘The Romantic Programme and the Reception of Cell Theory in Britain’, *Journal of the History of Biology*, 1984, 17, 13–48.

27 John Hughes Bennett, a keen advocate for the microscope in scientific investigation, was ambivalent over its diagnostic utility in cases of malignancy, ‘The microscope alone—that is, independently of all other kind of observation—can seldom determine in the living subject the presence or absence of Cancer’. John Hughes Bennett, *On Cancerous and Cancroid Growths* (Edinburgh: Sutherland and Knox, 1849), vii.

28 For example, the case of six-year-old Susannah Foster, treated for cancer of the eye at University College Hospital in 1853. John Zachariah Laurence, *The Diagnosis of Surgical Cancer*, 2 (London: John Churchill, 1858), 26.

29 Maurice Henry Collis, *On the Diagnosis and Treatment of Cancer and the Tumours Analogous to It* (London: John Churchill, 1864), 2.

30 Bennett, *On Cancerous and Cancroid Growths*, vii.

31 G. Ernest Herman, ‘An Address on the Early Diagnosis of Cancer of the Cervix Uteri’, *BMJ*, 1894, 1, 1009–12, 1011.

32 Edward Higgs, ‘Registrar General’s Reports for England and Wales, 1838–1858’, Online Historical Population Reports, accessed 13 October 2016, <http://histpop.org/>.

33 Ian Hacking, *The Taming of Chance* (Cambridge: Cambridge University Press, 1990).

34 ‘Statistical Nosology’, *Fourth Annual Report of the Registrar-General* (England, 1840–41), 93–105.

35 John M. Eyler, ‘The Conceptual Origins of William Farr’s Epidemiology: Numerical Methods and Social Thought in the 1830s’ in Abraham M. Lilienfield, ed., *Times, Spaces, and Persons* (Baltimore, MD: Johns Hopkins Press, 1980), 1.

36 Edward Higgs, ‘The Annual Report of the Registrar-General, 1839–1920: A Textual History’, in Eileen Magnello and Anne Hardy, eds, *The Road to Medical Statistics* (Amsterdam: Rodopi, 2002), 55.

37 ‘Deaths from several zymotic and other causes, and inquest cases, in the divisions, counties, and districts of England’, *Forty-second Annual Report of the Registrar-General* (England, 1879), 186–97.

38 These figures are derived from the forty-second annual report and replicate their language: ‘of all ages’. However, it is worth noting that the vast majority of the cases included in the figures would have been adult or elderly men and women. In the 1870s, only 426 deaths in England and Wales among those younger than fifteen were attributed to cancer. *Ibid*.

39 ‘Introduction’, *Forty-Second Annual Report*, xxx.

40 Hugh P. Dunn, ‘An Inquiry into the Causes of the Increase of Cancer’, *BMJ*, 1883, 1, 708–10, 708.

41 The social body, as elaborated by Michel Foucault, Mary Poovey, and others, refers to the metaphorical description of the population as a unified and specifically corporeal whole. Mary Poovey, *Making a Social Body: British Cultural Formation, 1830-1864* (Chicago: Chicago University Press, 1995).

42 Dunn, ‘An Inquiry into the Causes of the Increase of Cancer’, 708.

43 ‘Malaria a Cure for Cancer’, *The New York Times*, 7 April 1902.

44 Frank A. Barrett, ‘Alfred Haviland’s Nineteenth-Century Map Analysis of the Geographical Distribution of Diseases in England and Wales’, *Social Science and Medicine*, 1998, 46, 767–81, 768.

45 Alfred Haviland, *Climate, Weather, and Disease: Being a Sketch of the Opinions of the Most Celebrated Ancient and Modern Writers* (London: J. Churchill, 1855); *The Sanitary Regulations of Ancient Rome* (London: John Churchill, 1855).

46 Haviland, *The Geographical Distribution of Heart Diseases and Dropsy, Cancer in Females and Phthisis in Females, in England and Wales*; Haviland, *The Geographical Distribution of Disease in Great Britain*, 2nd edn (London: Swan Sonnenschein, 1892).

47 Haviland, *Geology in Relation to Sanitary Science* (London: G. Norman and Son, 1879); Haviland, *Scarborough as Health Resort: Its Physical Geography, Geology, Climate & Vital Statistics* (London: Adams Hamilton, 1883).

48 Haviland, *Climate, Weather and Disease*, vii.

49
*Ibid*., 5

50 German physician L. L. Finke produced a world map of disease in 1792: F. A. Barrett, ‘Ginke’s 1792 Map of Human Diseases: The First World Disease Map?’, *Social History of Medicine*, 2000, 50, 915–21.

51 Gregg Mitman and Ronald L. Numbers, ‘From Miasma to Asthma: The Changing Fortunes of Medical Geography in America’, *History and Philosophy of the Life Sciences*, 2003, 25, 391–412, 399.

52 By this I mean that anti-contagionism, rather than the complexity and nuance of eighteenth-century aetiological frameworks, found a champion in Chadwick and his allies.

53 Haviland, *The Geographical Distribution of Diseases in Great Britain*, 2nd edn, 5.

54
*Ibid*.

55 Koch, *Disease Maps*, 132.

56 John Snow, *On the Mode of Communication of Cholera*, 2nd edn (London: John Churchill, 1855).

57 Michael Worboys, *Spreading Germs: Disease Theories and Medical Practice in Britain, 1865**–**1900*, (Cambridge: Cambridge University Press, 2000), 23. For a full analysis of Chadwick’s understanding of disease aetiology see Christopher Hamlin, *Public Health and Social Justice in the Age of Chadwick: Britain, 1800**–**1854* (Cambridge: Cambridge University Press, 2008); and ‘Providence and Putrefaction: Victorian Sanitarians and the Natural Theology of Health and Disease’, *Victorian Studies*, 1985, 28, 381–411.

58 Gilbert, *Mapping the Victorian Social Body*, 10.

59 J. B. Harley, *The New Nature of Maps* (Baltimore, MD: Johns Hopkins University Press, 2006), 7.

60 Gilbert, ‘The Victorian Social Body and Urban Cartography’, 15.

61
*Ibid*., 15.

62 Quoted in Koch, *Disease Maps: Epidemics on the Ground*, 250.

63 Haviland, *Climate, Weather and Disease*, 5.

64 Haviland, *The Geographical Distribution of Diseases*, 2nd edn, 8.

65 Haviland, ‘Abstracts of Lectures on the Geographical Distribution of Disease in England and Wales’, I, *BMJ*, 1870, 2, 453–4, 453.

66 Haviland, *Scarborough as a Health Resort*, 7.

67 Haviland, *A Paper on the Influence of Clays and Limestones on Medical Geography*, 5.

68 In recent years, the production of scale has been scruitinised as a political-economic process. Rather than taking particular scales of social and cultural activity for granted (such as the nation state), these scholars are gesturing towards ways in which scale was contested and constructed. ‘Scale’, in Linda McDowell and Joanne P. Sharp, eds, *A Feminist Glossary of Human Geography* (London: Arnold, 1999), 242.

69 Haviland, ‘Abstracts of Lectures on the Geographical Distribution of Disease in England and Wales’, II, 573.

70 Elisha Bartlett, *An Essay on the Philosophy of Medical Science* (Philadelphia: Lea & Blanchard, 1844), 140.

71 Haviland, ‘Abstracts of Lectures on the Geographical Distribution of Disease in England and Wales’, II, *BMJ*, 1870, 2, 573–75, 573 [emphasis added].

72 Historian Laura Otis draws on the work of George Lakoff and Mark Johnson to illuminate the role of metaphors in scientific writing. She argues that metaphors do not ‘express’ scientists … they are the ideas’. Laura Otis, ‘The Metaphoric Circuit: Organic and Technological Communication in the Nineteenth Century’, *Journal of the History of Ideas*, 2002, 63, 105–128, 127.

73 Walter Hayle Walshe, *The Nature and Treatment of Cancer* (London: Taylor and Walton, 1846), 124.

74 This had much in common with the motivations for anatomical atlases. Daston and Galison have shown that atlases ‘standardised’ and represented ‘a dynamic, organic phenomenon that might differ from person to person, and moment to moment’. They transform the infinite varieties of health and ill-health into ‘characteristics’. Lorraine Daston and Peter Galison, *Objectivity* (New York: Zone Books, 2007).

75 Haviland, ‘Abstracts of Lectures on the Geographical Distribution of Disease in England and Wales’, II, 574.

76 Haviland, *The Geographical Distribution of Diseases in Great Britain*, 2nd edn, 10.

77 For the first quote in this sentence, see *ibid.*, 10; for the second *i**bid*., 11.

78 While he only mapped female cancer, Haviland argued that, ‘the two distributions of cancer among males and females are practically of the same species’ and that the images would look much the same. Haviland, *The Geographical Distribution of Diseases in Great Britain*, 2nd edn, 307.

79 For the first use of chloropleth mapping, see Gilbert, *Mapping the Victorian Social Body*, 11. The term ‘cartographic objective’ is to be found in Karen S. Pearson, ‘The Nineteenth-Century Colour Revolution: Maps in Geographical Journals’, *Imago Mundi*, 1980, 32, 9–20, 9.

80
*Ibid*.

81 Haviland, *The Geographical Distribution of Diseases in Great Britain*, 2nd edn, viii.

82 The idea that the Thames was pathological was not specific to Haviland. However, for other commenters the river was made toxic by human excrement and waste.

83 Haviland, *A Paper on the Influence of Clays and Limestones on Medical Geography*, 3.

84
*Ibid*, 8. It is worth noting that mapmaking (and the concerns of sanitation and public health) were also crucial for interrogating disease in the British colonies. See, Pamela K. Gilbert, ‘Mapping Colonial Disease: Victorian Medical Cartography in British India’, in George Sebastian Rousseau, Miranda Gill, David Haycock and Malte Herwig, eds, *Framing and Imagining Disease in Cultural History*, (Basingstoke: Palgrave Macmillan, 2003).

85
*Ibid*., 9.

86 Haviland, ‘Abstracts of Lectures on the Geographical Distribution of Disease in England and Wales’, II, 574.

87 Cholera has long been associated with the environment. For example, the German physician Freidrich Schnurrer identified a geographical cause for the disease in the first half of the nineteenth century.

88 Jacob Steere-Williams, ‘Performing State Medicine During its “Frustrating” Years: Epidemiology and Bacteriology at the Local Government Board, 1870–1900’, *Social History of Medicine*, 2014, 28, 82–107, 103.

89 Charles H. Moore, *The Antecedents of Cancer* (London, 1865), iv.

90
*Ibid*, 36.

91
*Ibid*, 37.

92
*Ibid*, 40.

93
*Ibid*, 38.

94
*Ibid*., 43.

95 He is cited by Haviland and many other mapmakers in the nineteenth century.

96 For an analysis of Collective Investigation more broadly, see Harry Marks, ‘“Until the Sun of Science … the true Apollo of Medicine has risen”: Collective Investigation in Britain and America, 1880–1910’, *Medical History*, 2006, 50, 147–66.

97 Isambard Owen, ‘Reports of the Collective Investigation Committee of the British Medical Association. Geographical Distribution of Rickets, Acute and Subacute Rheumatism, Chorea, Cancer, and Urinary Calculus in the British Islands’, *BMJ*, 1899, 1, 113–16, 114.

98
*Ibid*.

99 Wilfred Watkins-Pitchford, ‘Light, Pigmentation, and New Growth’, *BMJ*, 1909, 2, 442–3, 443.

100 P. W. Hislop and P. Clennell Fenwick, ‘Cancer in New Zealand’, *BMJ*, 1909, 2, 1222–5.

101 ‘Cancer in Relation To The Dwelling’, *BMJ*, 1898, 2, 1571–2, 1571.

102 E. Lloyd Jones, ‘The Topographical Distribution of Cancer’, *BMJ*, 1899, 1, 813–15, 814.

103
*Ibid*.

104 James Braithwaite, ‘Cancer Mortality’, *BMJ*, 1903, 1, 1289.

105 Alexander Urquhart, ‘Notes on Recent Cancer Mortality In The Thames Valley, *BMJ*, 1904, 1, 825–6, 825.

106
*Ibid*.

107 ‘Soil and Stomach Cancer’, *BMJ*, 1965, 1, 1–2, 1.

108 Henri Jouve, ‘These pour Le Doctorat en Médecine sur La Topographie et La Contagion du Cancer’ (Paris, 1897), 13.

109 Quoted in, Haviland, *The Geographical Distribution of Diseases in Great Britain*.

110
*Ibid*.

111 Koch, ‘Social Epidemiology as Medical Geography’, 197

112 Haviland’s decision to map cancer at over 35 years was significant. He thought that removing younger women from his sample would make the data more accurate, and the correlations more visually clear, because he considered this older period of life as, ‘an age when Cancer is most fatal’. Haviland, *The Geographical Distribution of Diseases in Great Britain*, 2nd edn, 300.

113 Haviland, *A Paper on the Influence of Clays and Limestones on Medical Geography; illustrated by the Geographical Distribution of Cancer among Females, in England and Wales* (London: John Bale & Sons, 1891), 3.

114 ‘The Defeated Ennerdale Railway’, *The Spectator*, 21 July, 1883, 929.

115
*Ibid*.

116 ‘Court and Fashionable’, *John Bull*, 20 July, 1844, 456.

117 See Yi-Fu Tuan’s ideas of ‘topophilia’ and ‘topophobia’. Yi-Fu Tuan, *Space and Place: The Perspective of Experience* (Minneapolis: University of Minnesota Press, 1977).

118
*The Fifth Annual Report of the Registrar-General*, 408.

119 Hugh P. Dunn, ‘Report of the Registrar-General’, *The Popular Science Monthly*, 1885, XXXVI, 689.

120 Nascent in this is the now-prevalent idea that cancer is a pathology of progress. See, Charles E. Rosenberg, ‘Pathologies of Progress: The Idea of Civilization as Risk’, *Bulletin of the History of Medicine*, 1998, 72, 714–30. Associations between various pathologies and civilisation were common in the *fin de siècle*. For example, in 1909, Iwan Bloch held that there is an intimate connection between civilization and ‘syphilization’. Iwan Bloch, *The Sexual Life of Our Time in its Relations to Modern Civilization* (London: Rebman, 1909), 384.

121 Hugh P. Dunn, ‘An Inquiry into the Causes of the Increase of Cancer (Continued)’, *BMJ*, 1883, 1, 761–3, 762.

122 D. Heron, *On the Relation of Fertility in Man to Social Status etc*., (London: Dulau and Co., 1906).

123 The period between 1860 and 1914 witnessed a dramatic fall in fertility in Britain and awareness of these developments prompted widespread anxiety and introspection. Simon Szreter, *Fertility, Class and Gender in Britain, 1860**–**1940* (Cambridge: Cambridge University Press, 1996).

124 Instead, cancer was conceptualised as a product of old age. In the 1870s, surgeon and pathologist James Paget claimed, ‘Cancer is a disease of degeneracy, the frequency of which increases as the years increase.’ With the increasing anxiety over rising cancer rates after *c*.1880, ‘the peculiar age-incidence’ of the disease attracted new attention. Quoted in Hugh P. Dunn, ‘An Inquiry into the Causes of the Increase of Cancer (Continued)’, *BMJ*, 1883, 1, 761. Nineteenth-century observers observed that cancer was a disease of the old, and many speculated that its increasing and varying incidence could be a product of increasing and varying life expectancy. Director of the Imperial Cancer Research Fund, E. F. Bashford argued that the cancer-causing qualities of civilisation were, paradoxically, in its ability to procure and maintain long life. E. F. Bashford, ‘An Address Entitled Are the Problems of Cancer Insoluble?’, *BMJ*, 1905, 2, 1509.

125 Frank Bushnell and F. Cavers, ‘Structural Links in Malignant Growths’, *BMJ*, 1904, 1, 1005–6, 1005.

126 The *BMJ* wrote, ‘The cancer cells spread … radially through the circular muscular coat to deeper plexuses, where they may travel for some distance.’ ‘Intramural Spread of Cancer of the Rectum’, *BMJ*, 1913, 1, 463–4, 463.

127 T. W. Nunn, ‘Diagram Map showing Geographical Distribution of the Place of Birth of the Cases enumerated in accompanying Table’, *On Cancer of the Breast* (London: J. & A. Churchill, 1882), 167.

## Notes

Agnes Arnold-Forster is a Postdoctoral Research Fellow on the Wellcome Trust project, ‘Surgery & Emotion’, based at the University of Roehampton. She completed her AHRC-funded PhD on the history of cancer in nineteenth-century Britain at King's College London in 2017. Her current research interests include surgery, medicine and nursing; gender, emotions and professional identity; and cultural representations of the National Health Service.

