# Mapmaking and Mapthinking: Cancer as a Problem of Place in Nineteenth–century England

**DOI:** 10.1093/shm/hkaa044

**Published:** 2020-06-11

**Authors:** Agnes Arnold-Forster

*Mapmaking and Mapthinking: Cancer as a Problem of Place in Nineteenth–century England*

by Agnes Arnold-Forster

*Social History of Medicine* 2018, hky059, https://doi.org/10.1093/shm/hky059

In the original article, the caption for Figure 3 was incorrect stating: “Cholera Map of the Metropolis, 1849, Exhibited in the Registration Districts 1850, Wellcome Library, London”.

This has been amended to reflect the correct caption of: “Alfred Haviland, The Geographical Distribution of Cancer (Females), Divisions, 1851–1860, Wellcome Library, London”

